# Intact colonic K_C__a_1.1 channel activity in KCNMB2 knockout mice

**DOI:** 10.14814/phy2.13179

**Published:** 2017-03-14

**Authors:** Casper K. Larsen, Helle A. Praetorius, Jens Leipziger, Mads V. Sorensen

**Affiliations:** ^1^ Department of Biomedicine, Physiology HealthAarhus UniversityAarhus CDenmark; ^2^ Aarhus Institute for Advanced Studies (AIAS)Aarhus University. Hoegh‐GuldbergsAarhus CDenmark

**Keywords:** BK channel, K^+^ Homeostasis, K^+^ secretion, K_C__a_1.1, K_C__a_1.1 beta2 subunit, LRCC26, Maxi K^+^ channel

## Abstract

Mammalian potassium homeostasis results from tightly regulated renal and colonic excretion, which balances the unregulated dietary K^+^ intake. Colonic K^+^ secretion follows the pump‐leak model, in which the large conductance Ca^2+^‐activated K^+^ channel (K_C__a_1.1**)** is well established as the sole, but highly regulated apical K^+^ conductance. The physiological importance of auxiliary *β* and *γ* subunits of the pore‐forming *α*‐subunit of the K_C__a_1.1 channel is not yet fully established. This study investigates colonic K^+^ secretion in a global knockout mouse of the K_C__a_1.1‐*β*2‐subunit (KCNMB2^−/−^), which apparently is the only *β*‐subunit of the colonic enterocyte K_C__a_1.1 channel. We can report that: (1) Neither K_C__a_1.1 *α*‐ nor the remaining *β*‐subunits were compensatory transcriptional regulated in colonic epithelia of KCNMB2^−/−^ mice. (2) Colonic epithelia from KCNMB2^−/−^ mice displayed equal basal and ATP‐induced K_C__a_1.1‐mediated K^+^ conductance as compared to KCNMB2^+/+^. (3) K^+^ secretion was increased in KCNMB2^−/−^ epithelia compared to wild‐type epithelia from animals fed an aldosterone‐inducing diet. (4) Importantly, the apical K^+^ conductance was abolished by the specific blocker of K_C__a_1.1 channel iberiotoxin in both KCNMB2^+/+^ and KCNMB2^−/−^ mice. Recently a novel family of auxiliary *γ*‐subunits of the K_C__a_1.1 channel has been described. (5) We detected the *γ*1‐subunit (LRRC26) mRNA in colonic epithelia. To investigate the physiological role of the *γ*1‐subunit of K_C__a_1.1 channels in colonic K^+^ secretion, we acquired an LRRC26 knockout mouse. (6) Unexpectedly, LRRC26 mice had a perinatal lethal phenotype, thus preventing functional measurements. On this basis we conclude that colonic K^+^ secretion is intact or even increased in mice lacking the *β*2‐subunit of K_C__a_1.1 channel complex despite no additional compensatory induction of K_C__a_1.1 *β*‐subunits.

## Introduction

Whole body K^+^ homeostasis is achieved by matching renal and colonic K^+^ excretion to dietary K^+^ intake. In the murine distal colon, K^+^ secretion takes place in the colonic crypts (Sorensen et al. 2011). According to the pump‐leak model, K^+^ enters the crypt cells via the basolateral Na^+^/K^+^‐ATPase or the Na^+^/K^+^/2Cl^−^‐cotransporter (NKCC1) and exits over the luminal membrane via the K_Ca_1.1 channel complexes (Sausbier et al. 2006). The K_Ca_1.1 channel consists of pore‐forming *α*‐subunits and regulatory *β*‐subunits, of which there are four different isoforms (*β*1–4) (Orio et al. 2002; Lippiat et al. 2003). The different *β*‐subunit isoforms confer different activation and inactivation properties to the K_Ca_1.1 channel, but all increases the Ca^2+^ sensitivity of the channel, thus facilitating channel activity (Brenner et al. 2000a; Lippiat et al. 2003; Orio and Latorre 2005). The *α*‐subunit of the K_Ca_1.1 channels is ubiquitously expressed. Tissue specific coexpression of the different *β*‐subunits is an important way, in which K_Ca_1.1 channel function can be tailored to cell type specific functions. In the murine distal colonic epithelium, K_Ca_1.1 channels are composed exclusively of *α*‐ and *β*2‐subunits (Sorensen et al. 2008).

The *β*2‐subunit is unique among the K_Ca_1.1 *β*‐subunits as it inflicts both activating and inactivating properties to the K_Ca_1.1 channel (Lippiat et al. 2003; Orio and Latorre 2005). Coexpression of *α*‐ and *β*2‐subunits increase open probability of the channel by increasing Ca^2+^‐sensitivity of the channel, when compared to channels composed of *α*‐subunits only (Lippiat et al. 2003; Orio and Latorre 2005). On the other hand, *β*2‐subunits mediate rapid inactivation of the K_Ca_1.1 channel via an N‐terminal ball‐and‐chain domain on the *β*2‐subunits (Bentrop et al. 2001) and decrease surface expression of the *α*‐subunit. The latter is suggested to be mediated by an endocytic sorting signal in the *β*2‐subunit (Zarei et al. 2007). Because of the dual nature of *β*2‐mediated regulation of the K_Ca_1.1 channel, it is difficult to predict the overall effect of removal of *β*2‐regulation of K_Ca_1.1 channels in the murine distal colon.

In this study, we use a mouse model with a global deletion of the *β*2‐subunit (KCNMB2^−/−^) of the K_Ca_1.1 channel to investigate the functional role of the *β*2‐subunit in distal colonic K^+^ secretion. In accordance with our previous work, we found that murine distal colonic epithelial cells express *α*‐ and *β*2‐subunits (Sorensen et al. 2008). K_Ca_1.1 channels that are composed of *α*‐subunits only require substantial membrane depolarization to open (Orio et al. 2002; Lippiat et al. 2003). This raises the question as to how distal colonic K_Ca_1.1 channel activity is supported in KCNMB2^−/−^ mice. One answer could be provided by the family of *γ*‐subunits (*γ*1‐4, encoded by the genes LRRC26, LRRC52, LRRC55, and LRRC38, respectively) recently described for the K_Ca_1.1 channel. These *γ*‐subunits similar to the *β*‐subunits modulate K_Ca_1.1 channel function (Yan and Aldrich 2010, 2012). LRRC26 mRNA expression has been reported in whole human colon (Yan and Aldrich 2012), and we demonstrate mRNA expression of LRRC26 in the distal colonic epithelium. To address whether *γ*1‐subunits support K_Ca_1.1 channel activity in the murine distal colon, we acquired a global LRRC26 knockout mouse. LRRC26^−/−^ mice did not survive after birth, precluding a description of distal colonic K^+^ secretion in these mice.

## Materials and Methods

### Mice, diets, and housing

The KCNMB2 strain was generated in the 129/SvEvBrd x C57BL/6 background by the Texas Institute of Genomic Medicine (College Station, TX). The *KCNMB2* gene was disrupted by truncation of exon 3 and deletion of exons 4 and 5. Mice were bred from heterozygous families, yielding KCNMB2^+/+^ and KCNMB2^−/−^ littermates. Mice were genotyped as described previously (Larsen et al. 2016). The LRRC26 strain was generated in the C57BL/6NTac background by KOMP, Davis, CA. The LRRC26 gene was disrupted by insertion of a cassette encoding the LacZ and neomycin resistance genes. Mice were bred from heterozygous breeding families. Genotyping was performed with the primers WT‐Fw: 5′‐ATATTGGAACTGCCCCTTCTAAATCCC‐3′, WT‐Rev: 5′‐GTTCTCCCTCAGATCTAGGTATAGCAGC‐3′, KO‐Fw: 5′‐GCAGCCTCTGTTCCACATACACTTCA‐3′, and KO‐Rev: 5′‐TACATGACTAGCTCTCCCAGTGTCC‐3′. To determine whether LRRC26 ablation resulted in early termination of embryos, late gestational fetuses were isolated from two pregnant females.

The mice were given ad libitum access to food and water, and kept in cages with not more than four mice per cage. Mice were fed either a normal diet (Altromin 1310, 10 g K^+^/kg, 2 g Na^+^/kg) or an aldosterone‐inducing high K^+^/low Na^+^ diet (Altromin 1350, 50 g K^+^/kg, 0.2 g Na^+^/kg). Both male and female mice were used at the age between 6 and 16 weeks. All experiments were performed in accordance with the Danish legislation on the protection of animals.

### Isolation of distal colonic epithelium

The most distal 4 cm of the colon was dissected free and feces content was flushed out with ice‐cold Ca^2+^‐free Ringer. Colon was inverted by inserting a glass rod into the colon via the anus, tying a suture around colon and glass rod at the proximal end and retracting the glass rod. The distal 2 cm of the inverted colon was tied at both ends with suture and the sack was filled with Ca^2+^‐free Ringer and incubated in Ca^2+^‐free Ringer at 37°C for 10 min while being slowly shaken. After incubation, the inverted colonic sacks were vigorously shaken by hand, causing the epithelium to detach from the colon. The colonic sacks were discarded, and the isolated colonic epithelia in the liquid phase were centrifuged at 10.392*g*, 4°C for 10 min, yielding pellets of epithelial cells. This preparation has previously been shown to be devoid of smooth muscle contamination (Sorensen et al. 2011).

### Semi‐quantitative RT‐PCR

RNA was isolated from distal colonic epithelia by RNEasy mini kits (Qiagen). Generation of cDNA was performed with Superscript III and Superase (Invitrogen), and semi‐quantitative RT‐PCR analysis was performed with Taqman Gene Expression Assays (Applied Biosystems) for KCNMA1 (Mm00516078_m1), KCNMB1 (Mm00466621_m1), KCNMB2 (Mm00511481_m1), KCNMB3 (Mm01292437_m1), KCNMB4 (Mm00465684_m1), LRRC26 (Mm00525100_g1), LRRC38 (Mm01718430_m1), LRRC52 (Mm04204090_m1), LRRC55 (Mm02600578_m1), HPRT (Mm01545399_m1), and *β*‐actin (Mm00607939_s1). Relative mRNA expression was calculated using the 2^−∆Ct^‐method (Schmittgen and Livak 2008), the average *C*_t_ value for HPRT and *β*‐actin was used as reference *C*_t_ (Schmittgen and Livak 2008). All expression assays have been verified on positive expressing tissues in a previous publication from our group (Larsen et al. 2016).

### Ussing chambers

The most distal 4 cm of the colon was dissected free and feces were flushed out with ice‐cold Ringer solution. The most distal 2 cm of the colon was mounted in Ussing chambers with an aperture area of 0.238 cm^2^. The apical and basolateral compartments were perfused with 7.5 mL Ringer solution, bubbled with 5% CO_2_, 21% O_2_, 74% N_2_ and heated to 37°C by a heated water jacket. Experiments were performed in open‐circuit mode; transepithelial voltage (*V*_te_) was measured continuously. The transepithelial resistance was calculated from the size of voltage deflections induced by injection of current pulses of 25 *μ*A with a duration of 1 sec (Δ*V*_te_). These current pulses were injected continuously with a frequency of 0.2 Hz. Using Ohm's law, the equivalent short circuit current (*I*′_sc_) was calculated from the transepithelial voltage and resistance. Upon mounting of the tissue in the Ussing chambers, 5 *μ*mol/L of indomethacin was added to both the apical and basolateral compartments and 1 *μ*mol/L tetrodotoxin was added to the basolateral compartment to minimize prostaglandin‐mediated and neuronal stimulation of Cl^−^ secretion in response to the mechanical stimulation during the mounting procedure. After mounting, the tissue was allowed to stabilize for 10 min, whereupon 100 *μ*mol/L of amiloride was added to the apical compartment to inhibit ENaC‐mediated Na^+^ transport. Five minutes after amiloride application, K_Ca_1.1 channel activity was quantified as the change in *I*′_sc_ induced by luminal application of 5 mmol/L Ba^2+^ (added as BaCl_2_). Ba^2+^ is an unspecific K^+^ channel blocker, but can be used to quantify K_Ca_1.1 channel activity in the distal colon as the K_Ca_1.1 channel is the only apical K^+^ channel in this tissue (Sausbier et al. 2006). Experiments with iberiotoxin (IBTX) (Latoxan, Valence, France) were performed to ascertain that the Ba^2+^‐sensitive *I′*_sc_ in distal colonic epithelia from KCNMB2^−/−^ mice was in fact mediated by the K_Ca_1.1 channel. Due to the size of IBTX (4230 Da) and the localization of K_Ca_1.1 channels to the apical membrane of mucus covered distal colonic crypts (Sorensen et al. 2011), IBTX diffusion to the site of the K_Ca_1.1 channels is slow. Consequently, the effect of IBTX develops over a longer time (Sorensen et al. 2008, 2010), as opposed to the effects of amiloride and Ba^2+^, which occurs within 30 sec after application (Sausbier et al. 2006; Matos et al. 2007). We quantified the effect of 120 nmol/L luminal IBTX as the Ba^2+^ sensitive short circuit current 30 min after application of IBTX. K_Ca_1.1 channel activity can also be transiently stimulated by application of nucleotides to the apical compartment (Matos et al. 2005). ATP‐induced K_Ca_1.1 activation was studied by application of 100 *μ*mol/L ATP to the apical compartment. The maximal (peak) value of the transient changes in short circuit current was quantified.

### Solutions

Ussing chamber Ringer: 120 mmol/L NaCl, 0.4 mmol/L KH_2_PO_4_, 1.6 mmol/L K_2_HPO_4_, 1 mmol/L MgCl_2_, 5 mmol/L glucose, 1.3 mmol/L Ca‐gluconate, 2 mmol/L Na‐HEPES. pH was adjusted to 7.4, whereupon 25 mmol/L NaHCO_3_ was added to yield a pH of 7.4 when the solution was bubbled with the 5% CO_2_, 21% O2, 74% N_2_ gas mixture.

Ca^2+^‐free Ringer: 127 mmol/L NaCl, 5 mmol/L KCl, 1 mmol/L MgCl_2_, 5 mmol/L Na‐pyruvate, 10 mmol/L Na‐HEPES, 5 mmol/L Na‐EDTA (C_10_H_16_N_2_O_8_), 5 mmol/L glucose.

### Statistics

The assumption of normal distribution of data was tested by the Kolmogorov–Smirnov test. Data were analyzed by Student's *t*‐test for comparison of two experimental groups or by one‐way ANOVA for multiple comparisons. Statistical analysis was performed with the GraphPad Prism software (version 4.02). The Mendelian genotype distributions were tested by a chi‐square test for Mendelian ratios by the use of the web page http://www.ihh.kvl.dk/htm/kc/popgen/genetik/applets/ki.htm. The calculated chi‐square values were evaluated by the use of a table of percentage point of chi‐square distribution.

## Results

### Expression of K_Ca_1.1 α‐ and β‐subunits in the distal colonic epithelium

**Figure 1:**
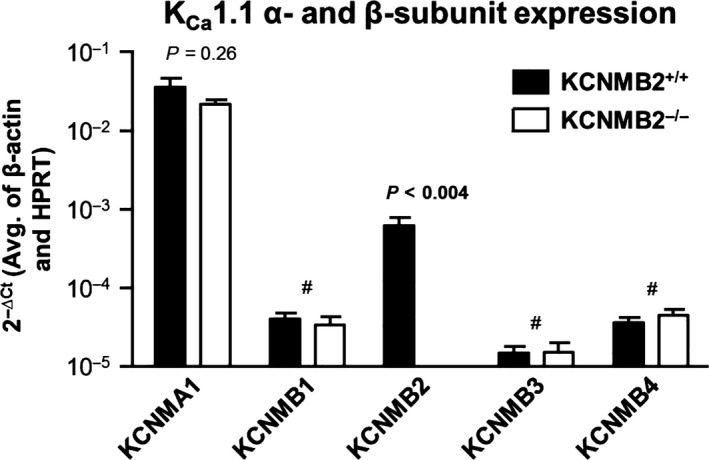
mRNA expression of BK channel *α*‐ and *β*‐subunits in isolated colonic crypts from KCNMB2^+/+^ and KCNMB2^−/−^ mice relative to reference genes (*β*‐actin and HPRT). KCNMA1 (*C*_t_~26) and KCNMB2 (*C*_t_~30) were detectable in crypts from KCNMB2^+/+^. In crypts from KCNMB2^−/−^ mice mRNA of KCNMB2 was not detectable whereas mRNA from crypts of KCNMA1 similar to KCNMB2^+/+^. # denotes that KCNMB1, KCNMB3 and KCNMB4 were detectable but in irrelevant quantities (*C*_t_ > 38). Data are shown as means ± SEM,* n* = 6 in all measurements.

We used semi‐quantitative rt‐PCR to study expression of *α*‐ and *β*‐subunits of the K_Ca_1.1 channel in preparations of distal colonic epithelium. We confirmed our previous observation (Sorensen et al. 2008) that only the *β*2 subunit is expressed at a significant level in the tissue (Fig. 1). *β*2 mRNA was undetectable in preparations from KCNMB2^−/−^ mice. No compensatory upregulation was observed in either the *α*‐subunit or in any of the remaining *β*‐subunits, although mRNA expression of *β*1, *β*3, and *β*4 was too low (C_*t* _> 38) to allow quantitative comparison of expression between genotypes.

### Increased aldosterone stimulated colonic K_Ca_1.1 activity in KCNMB2^−/−^ mice

**Figure 2:**
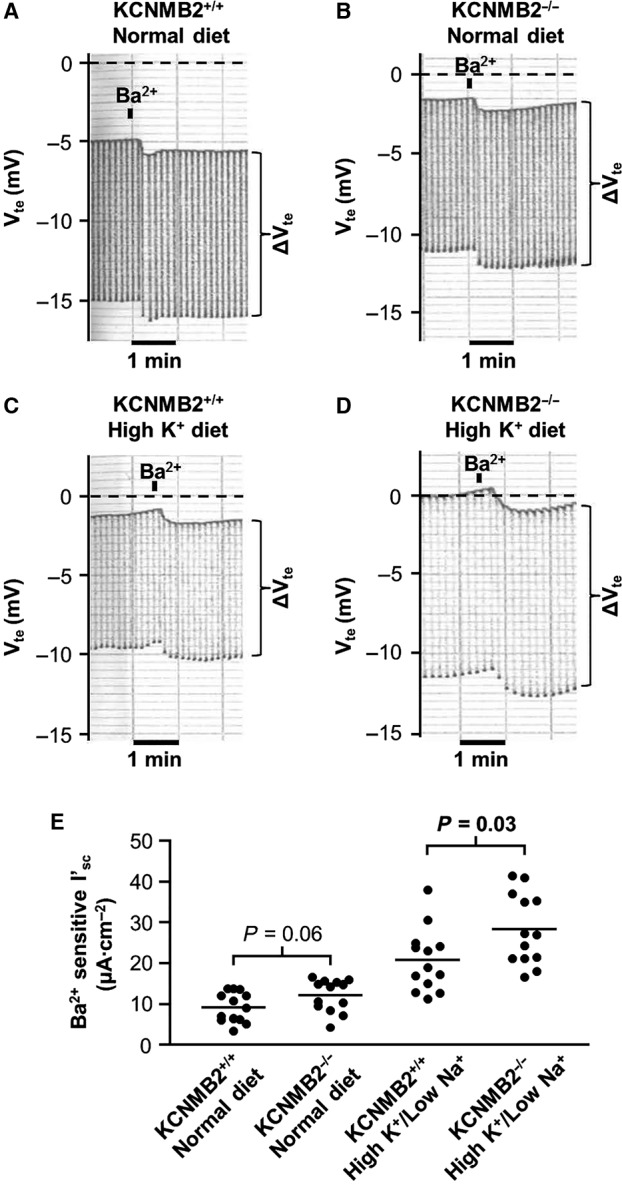
Barium sensitivity in isolated colonic mucosa from KCNMB2^+/+^ and KCNMB2^−/−^ mice. (A–D) shows original representative recordings of barium‐sensitive transepithelial voltages (*V*_te_) and resistance (Δ*V*_te_) in tissues from KCNMB2^+/+^ and KCNMB2^−/−^ mice kept on control or aldosterone‐augmenting diets. (E) summarizes the calculated Ba^2+^‐sensitive short circuit currents (*I*_sc_) in each of the four experimental groups. Horizontal lines represent mean values, *n* = 13 in each group. Note that Ba^2+^‐sensitive *I*_sc_ is similar in all genotypes on control diet but statistically significantly augmented in the KCNMB2^−/−^ as compared to KCNMB2^+/+^ on the aldosterone‐augmenting diet.

The effect of KCNMB2 ablation on distal colonic K_Ca_1.1 channel activity was investigated in Ussing chambers. Basal K_Ca_1.1 channel activity was quantified as Ba^2+^‐sensitive *I*′_sc_ and measured to be 9.2 ± 1.0 *μ*A cm^−2^ (*n* = 13) in KCNMB2^+/+^ preparations and 12.2 ± 1.1 *μ*A cm^−2^ (*n* = 13) in KCNMB2^−/−^ preparations (Fig. 2A, B and E). Colonic K_Ca_1.1 channel activity is *per se* low in rodents kept on a normal diet, because of the low plasma aldosterone levels in animals on this diet. We speculated that differences in K_Ca_1.1 channel activity between KCNMB2^+/+^ and KCNMB2^−/−^ mice might be more visible if the K^+^ secretion was stimulated by an aldosterone‐inducing diet for 4 days. This maneuver increased plasma aldosterone and distal colonic ENaC activity, the latter measured as amiloride sensitive *I*′_sc_ (data presented in table 1). Interestingly, the K_Ca_1.1 channel activity was markedly increased in distal colonic epithelia from KCNMB2^−/−^ mice, compared to KCNMB2^+/+^ mice, when fed an aldosterone‐inducing high K^+^/low Na^+^ diet for 4 days. The Ba^2+^‐sensitive *I′*_sc_ was higher in the KCNMB2^−/−^ preparations (28.3 ± 2.4 *μ*A cm^−2^ [*n* = 13]) when compared with the KCNMB2^+/+^ preparations (20.8 ± 2.1 *μ*A cm^−2^ [*n* = 13]) from animals kept for 4 days on the aldosterone‐inducing high K^+^/low Na^+^ diet (Fig. 2C, D and E). In summary, Ba^2+^‐sensitive baseline colonic K_Ca_1.1 activity is not altered by deletion of KCNMB2 under normal dietary conditions, but is augmented in mice subjected to simultaneous dietary K^+^ loading and Na^+^ depletion.

**Table 1:** Plasma aldosterone and distal colonic amiloride sensitive *I*′_sc_ in KCNMB2^+/+^ and KCNMB2^−/−^ kept 4 days on an aldosterone‐inducing diet

Mouse genotype	Control diet		Aldosterone‐inducing diet	
	KCNMB2^+/+^	KCNMB2^−/−^	KCNMB2^+/+^	KCNMB2^‐/‐^
Plasma aldosterone (pg/mL)	343 ± 39 (*n* = 6)	596 ± 73 (*n* = 6)^b^	1756 ± 265 (*n* = 8)^a^	1850 ± 217 (*n* = 10)^a^
Amiloride‐sensitive *I*′_sc_ (*μ*A cm^−2^)	−19.6 ± 1.9 (*n* = 14)	−20.3 ± 3.0 (*n* = 6)	−389.0 ± 46.0 (*n* = 8)^a^	−569.4 ± 77.3 (*n* = 10)^a^

a *P* < 0.05 between same genotype on different diet.

b *P* < 0.05 between genotypes on same diet.

### Ba^2+^‐sensitive short circuit current is inhibited by specific K_Ca_1.1 channel blocker

**Figure 3:**
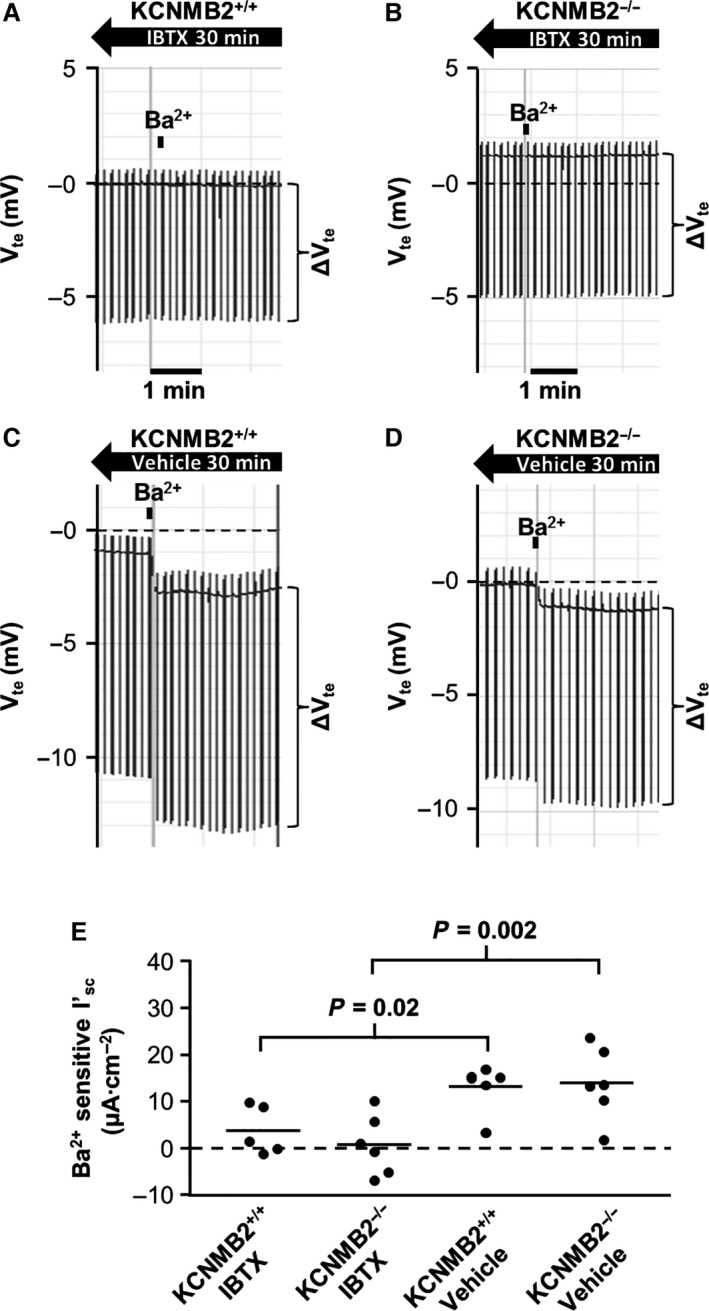
Barium sensitivity in isolated colonic mucosa from KCNMB2^+/+^ and KCNMB2^−/−^ mice pretreated 30 min with luminal (120 nmol/L) iberiotoxin (IBTX) or vehicle. (A–D) show original representative recordings of Ba^2+^‐sensitive transepithelial voltages (*V*_te_) and resistance (Δ*V*_te_) in tissues, pretreated with luminal IBTX or vehicle, from KCNMB2^+/+^ and KCNMB2^−/−^ mice. (E) summarizes the calculated Ba^2+^‐sensitive short circuit currents (*I*_sc_) in each of the four experimental groups. Horizontal lines represent mean values, *n* = 6 in each group. Note that Ba^2+^‐sensitive *I*_sc_ is almost abolished after pretreatment with IBTX.

To verify whether the Ba^2+^‐sensitive *I*′_sc_ in KCNMB2^−/−^ mice was in fact mediated by the K_Ca_1.1 channel, we performed Ussing chamber experiments, in which 120 nmol/L of the specific K_Ca_1.1 channel inhibitor IBTX was added to the luminal compartment. Thirty minutes after addition of IBTX or vehicle, BaCl_2_ (5 mmol/L) was added to the luminal perfusate to assess any Ba^2+^‐sensitive short circuit was measurable in the presence of IBTX. These experiments were performed with preparations from mice kept on the aldosterone‐inducing diet for 4 days. We measured a Ba^2+^‐sensitive *I*′_sc_ of 13.2 ± 2.0 *μ*A cm^−2^ (*n* = 6) in the KCNMB2^+/+^ vehicle time controls, whereas Ba^2+^‐sensitive *I*′_sc_ was reduced to 3.7 ± 2.3 *μ*A cm^−2^ (*n* = 5) in preparations pretreated with IBTX (Fig. 3). The same pattern was observed in KCNMB2^−/−^ preparations, here we measured 13.9 ± 3.2 *μ*A cm^−2^ (*n* = 6) in the vehicle time controls and 0.7 ± 2.6 *μ*A cm^−2^ (*n* = 6) in preparations pretreated with IBTX (Fig. 3). These data indicate that no additional K^+^ conductance other than the K_Ca_1.1 channel contributes to Ba^2+^‐sensitive *I*′_sc_ in KCNMB2^−/−^ mice.

### ATP‐induced K_Ca_1.1 activity was higher in colon from KCNMB2^−/−^ mice at high plasma aldosterone

**Figure 4:**
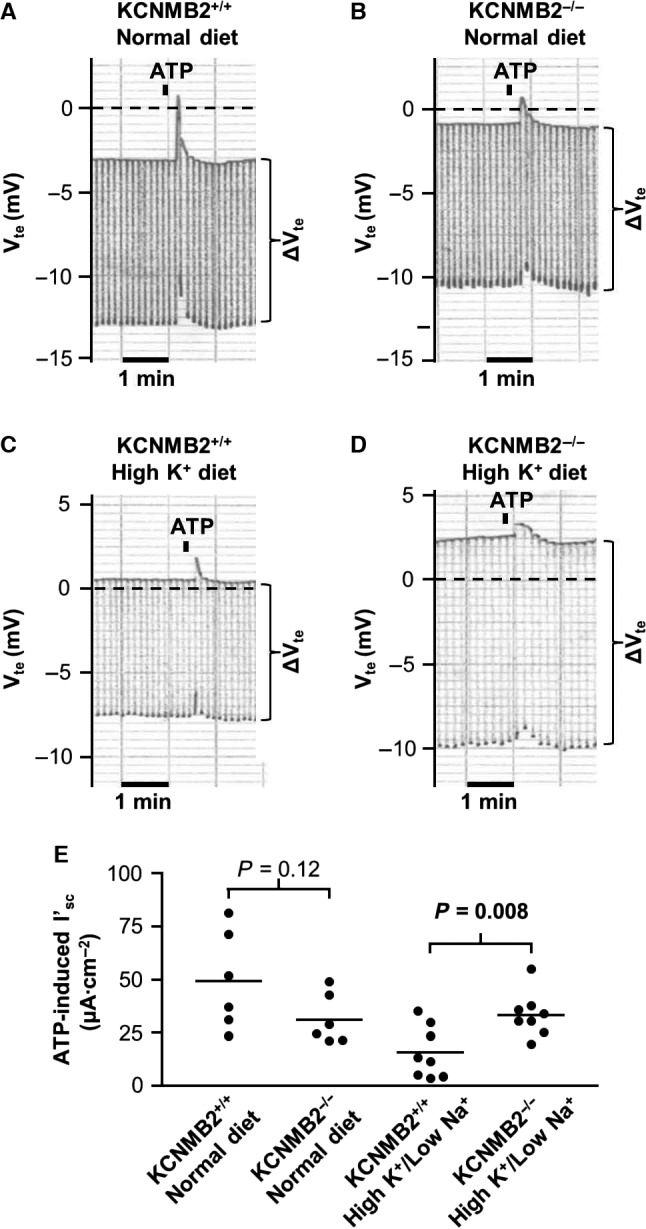
Effects of ATP stimulated on isolated colonic mucosa from KCNMB2^+/+^ and KCNMB2^−/−^ mice. (A–D) shows original representative recordings of luminal ATP effects on transepithelial voltages (*V*_te_) and resistance (Δ*V*_te_) in tissues from KCNMB2^+/+^ and KCNMB2^−/−^ mice kept on control or aldosterone‐augmenting diets. (E) summarizes the calculated ATP induced peak in short circuit currents (*I*_sc_) in each of the four experimental groups. Horizontal lines represent mean values, *n* = 6–7 in each group.

K_Ca_1.1 channel activity is stimulated by increased intracellular Ca^2+^ concentration ([Ca^2+^]_i_). The nucleotides ATP and UTP are known to cause reliable, transient increase in [Ca^2+^]_i_ in colonic epithelia via luminal P2Y receptors (Matos et al. 2005). Addition of ATP (100 *μ*mol/L) to the luminal perfusion of the Ussing chamber was followed by a transient lumen‐positive deflection in transepithelial voltage. This deflection has previously been shown to be caused by opening of apical K_Ca_1.1 channels (Sausbier et al. 2006). The ATP‐induced change in *I*′_sc_ was not different in preparations from the two genotypes (49.3 ± 9.4 *μ*A cm^−2^ (*n* = 6) in the KCNMB2^+/+^ and 31.2 ± 4.8 *μ*A cm^−2^ (*n* = 6) in the KCNMB2^−/−^) (Fig. 4A, B and E). In tissues from mice kept on the aldosterone‐inducing diet for 4 days, a significantly larger peak in ATP‐induced *I*′_sc_ was observed in the KCNMB2^−/−^ preparations than in KCNMB2^+/+^ preparations (33.4 ± 3.7 *μ*A cm^−2^ [*n* = 8] and 15.6 ± 4.3 *μ*A cm^−2^ [*n* = 8], respectively, Fig. 4C, D and E).

### Expression of K_Ca_1.1 γ‐subunits in the distal colonic epithelium

**Figure 5:**
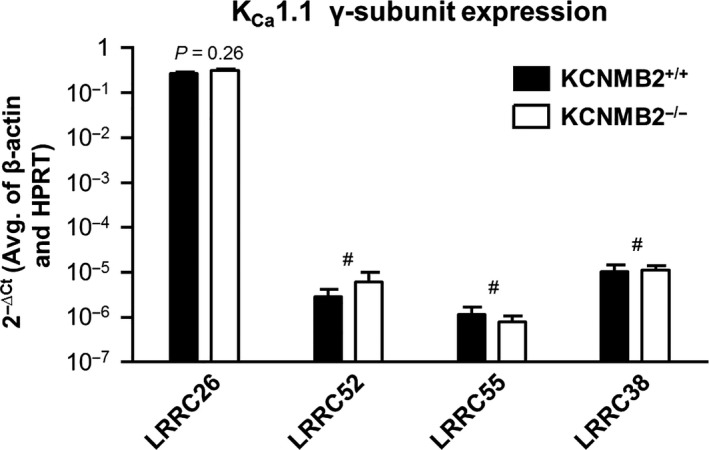
mRNA expression of BK channel *γ*‐subunits (LRRC) in isolated colonic crypts from KCNMB2^+/+^ and KCNMB2^−/−^ mice relative to reference genes (*β*‐actin and HPRT). LRRC26 was the only subunit detectable in relevant quantities (*C*_t_~25) in isolated colonic crypts. The level of LRRC26 mRNA was not dependent on the KCNMB2 gene. The remaining LRRC subunits were only detectable at very high *C*_t_ values. # denotes that mRNA was detectable but in irrelevant quantities (*C*_t_ > 38). Data are shown as means ± SEM,* n* = 6 in all measurements.

A new family of K_Ca_1.1 channel *γ*‐subunits, which modulate K_Ca_1.1 channel activity similarly to the *β*‐subunits, has recently been described, and mRNA expression of some of these *γ*‐subunits has been detected in whole human colon (Yan and Aldrich 2012). It is possible that the intact K_Ca_1.1 channel activity in KCNMB2^−/−^ mice could be attributed to expression of one or more of these *γ*‐subunits. We therefore performed semi‐quantitative rt‐PCR to determine whether any of the *γ*‐subunits were expressed in the murine distal colonic epithelium. We detected high mRNA expression of *γ*1 (LRRC26), whereas mRNA expression of *γ*2, *γ*3 and *γ*4 was absent or very low in colonic epithelium from KCNMB2^+/+^ mice (Fig. 5). There were no differences in distal colonic mRNA expression in any of the *γ*‐subunits between KCNMB2^+/+^ and KCNMB2^−/−^ mice (Fig. 5).

### Post‐natal lethality of LRRC26 mice

**Figure 6:**
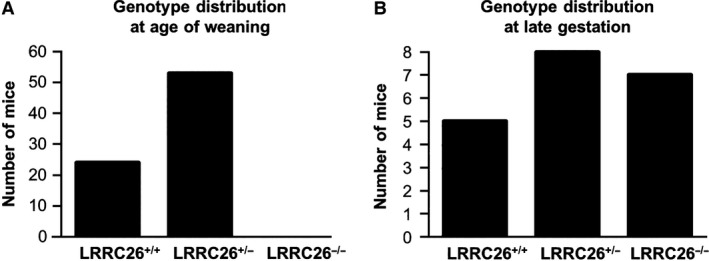
Genotype distribution in LRRC26 mice. (A) Age of weaning (~ 3 weeks). (B) late gestation (~ day 18).

To investigate whether the *γ*1‐subunit supports K_Ca_1.1 channel activity in the distal colon, we acquired a knock‐out mouse for the *γ*1‐subunit (LRRC26^−/−^). In total, we genotyped 77 pups from 10 litters at the time of weaning (day 21) and this resulted in 24 LRRC26^+/+^, 53 LRRC26^+/−^ and no LRRC26^−/−^ mice (Fig. 6A). These numbers fit significantly to a normal Mendelian distribution where the LRRC26^−/−^ mice were absent. The breeding families were checked for new off spring every morning, meaning that newborn pups were discovered before they were 24 h old. We did not observe any reduction in the number of pups in the litters between the first observation of pups and the day of weaning. To determine whether LRRC26 ablation resulted from early abortion of LRRC26^−/−^ embryos, we genotyped 20 late gestational fetuses (~day 18) from heterozygous breeding families. No apparent developmental defects were noticeable in any of the 20 fetuses, and the genotype distribution of the 20 fetuses were 5 LRRC26^+/+^, 8 LRRC26^+/−^, and 7 LRRC26^−/−^ (Fig. 6A). These data indicate that LRRC26 genotypes, as expected, shows a Mendelian distribution, but with a lethal phenotype of LRRC26^−/−^, which manifests during birth or shortly thereafter.

## Discussion

In this study, we describe the effect of KCNMB2 ablation on distal colonic K_Ca_1.1 channel activity. We confirm our observation, published in 2008, that the *β*2‐subunit of the K_Ca_1.1 channel is the only K_Ca_1.1 *β*‐subunit transcribed in the murine distal colon (Sorensen et al. 2008). Despite the lack of the only apparent *β*‐subunit in the distal colonic epithelium, preparations of the distal colon from KCNMB2^−/−^ mice displayed basal Ba^2+^‐sensitive *I*′_sc_, which was equal in size to that in KCNMB2^+/+^ preparations. In preparations from mice kept on an aldosterone‐inducing diet, the Ba^2+^‐sensitive *I*′_sc_ was increased in both genotypes, and this to even higher values in the KCNMB2^−/−^ tissues than in KCNMB2^+/+^ tissues.

On the physiological level, K_Ca_1.1‐mediated colonic K^+^ secretion is tightly regulated. A number of different receptor agonist acts as physiologically active secretagogues (Rechkemmer et al. 1996; Matos et al. 2005; Sorensen et al. 2010; Zhang et al. 2012). Luminal ATP has been shown to acutely stimulate colonic K^+^ secretion mediated via K_Ca_1.1 channels. The physiological purpose of this effect is not clear. It has been suggested that the increased ion and water secretion observed when gastrointestinal tract is stimulated by luminal nucleotides could be a host defense reaction (Leipziger 2003). In this study, the ATP‐induced *I*′_sc_ was comparable between distal colonic preparations from KCNMB2^−/−^ and KCNMB2^+/+^ mice. Interestingly, ATP‐induced *I*′_sc_ was increased in KCNMB2^−/−^ mice compared to KCNMB2^+/+^ mice when the animals were fed an aldosterone‐inducing diet for 4 days.

The observations that both basal and ATP‐stimulated K^+^ conductance is intact in the colon from KCNMB2^−/−^ mice could potentially be explained either by expression of other K^+^ channels or by intact K_Ca_1.1 channel activity in the absence of the *β*2‐subunit. It has been suggested that other K^+^ channels, in addition to K_Ca_1.1, may contribute to distal colonic K^+^ secretion in other mammalian species (Zhang et al. 2012). However, in the current study, apical application of the specific K_Ca_1.1 channel blocker IBTX, indisputably demonstrated that distal colonic K^+^ secretion also is mediated by K_Ca_1.1 channels in the absence of the KCNMB2 subunit. This is in line with numerous previous studies that finds the K_Ca_1.1 channel as the only important apical conductance for murine colonic K^+^ secretion (Sausbier et al. 2006; Sorensen et al. 2008, 2010). One could speculate that the physiological function of the K_Ca_1.1 channel, in the absence of KCNMB2^−/−^ subunits, was supported by one or some of the other *β*‐subunit (*β*1, *β*3 or *β*4). We used semi‐quantitative rt‐PCR to investigate the transcription level of the remaining *β*‐subunits. The *β*1, *β*3 or *β*4 subunits were all virtually undetectable in colonic epithelia from both KCNMB2^−/−^ and KCNMB2^+/+^ mice. This raises the question of what supports distal colonic K_Ca_1.1 activity in the absence of the *β*2‐subunit. A recently identified family of *γ*‐subunits, which are able to substantially modulate the K_Ca_1.1 channel in a manner similar to the *β*‐subunits (Yan and Aldrich 2010, 2012) may provide an answer to this question. We detected high mRNA expression of the *γ*1‐subunit in the distal colonic epithelium of both KCNMB2^+/+^ and KCNMB2^−/−^ mice. These data indicate that murine distal colonic K_Ca_1.1 channel activity could be regulated by both the *β*2‐ and the *γ*1‐subunit. Expression of a *γ*1‐subunit of the K_Ca_1.1 channel might contribute significantly to the physiological function of the K_Ca_1.1 channel. While increasing the Ca^2+^‐sensitivity of the K_Ca_1.1 channel, the *β*2‐subunit also causes rapid inactivation and reduced surface expression of the K_Ca_1.1 channel (Bentrop et al. 2001; Zarei et al. 2007). In the absence of *β*2‐subunit one could expect less inactivation and higher surface expression of the K_Ca_1.1 channel, which potentially could explain why K_Ca_1.1 activity was increased in the KCNMB2^−/−^ mice. It is unresolved whether *β*‐ and *γ*‐subunits of K_Ca_1.1 can regulate the channel simultaneously, although heterologous expression experiments indicate that *β*‐ and *γ*‐subunits interact with the *α*‐subunit at the same domain, and that *β*‐subunits may out‐compete *γ*‐subunits for interaction with *α*‐subunits when both are present (Yan and Aldrich 2012). However, the *γ*1‐subunit has been demonstrated to participate in regulation of the K_Ca_1.1 channel in isolated smooth muscle arterial cells (Evanson et al. 2014), in which the K_Ca_1.1 channels have previously been thought to be composed of *α*‐ and *β*1‐subunits (Brenner et al. 2000b; Pluger et al. 2000).

To investigate the role of the *γ*1‐subunit in murine distal colonic K^+^ secretion, we acquired a *γ*1‐knock‐out mouse (LRRC26^−/−^). Surprisingly, LRRC26 ablation led to a perinatal lethal phenotype. That a deletion of a K_Ca_1.1 channel *γ*1‐subunit resulted in a lethal phenotype was unexpected since mice with a global deletion of the pore‐forming *α*‐subunit of the K_Ca_1.1 channel complex are viable, but display severe motoric, neurologic, and endocrine disturbances (Ruttiger et al. 2004; Sausbier et al. 2004, 2005; Engel et al. 2006; Typlt et al. 2013). This suggests that the LRRC26 gene or gene product could have other cellular functions in addition to being an auxiliary subunit of the K_Ca_1.1 channel complex. The lethal phenotype of the LRRC26^−/−^ mice, rendered us were unable to perform experiments to address a possible role of the *γ*1‐subunit in the distal colonic K^+^ secretion.

In summary, KCNMB2^−/−^ mice did not display disturbed distal colonic K^+^ secretion, indicating that the *β*2‐subunit of the K_Ca_1.1 channel is not essential to support distal colonic K^+^ secretion. In fact, distal colonic K^+^ secretion was increased in KCNMB2^−/−^ mice compared to KCNMB2^+/+^ mice when animals were fed a high K^+^/low Na^+^ diet. The intact distal colonic K_Ca_1.1 activity in KCNMB2^−/−^ mice might have been supported by the *γ*1‐subunit of the K_Ca_1.1 channel, although the role of this subunit in distal colonic K^+^ secretion needs to be studied in a tissue specific LRRC26 knock‐out model.

## Conflict of Interest

There are no conflicts of interest to disclose.
